# Combined Anti-Adipogenic Effects of Hispidulin and *p*-Synephrine on 3T3-L1 Adipocytes

**DOI:** 10.3390/biom11121764

**Published:** 2021-11-25

**Authors:** Dahae Lee, Hee Jae Kwak, Byoung Ha Kim, Seung Hyun Kim, Dong-Wook Kim, Ki Sung Kang

**Affiliations:** 1 College of Korean Medicine, Gachon University, Seongnam 13120, Korea; pjsldh@gachon.ac.kr (D.L.); kimsh11@yonsei.ac.kr (S.H.K.); 2 College of Pharmacy, Yonsei Institute of Pharmaceutical Sciences, Yonsei University, Incheon 21983, Korea; moon3685@naver.com; 3 D. Nature Co., Ltd., Seongnam 13174, Korea; mot37@d-nature.co.kr; 4 Department of Pharmaceutical Engineering, Cheongju University, Cheongju 28530, Korea

**Keywords:** hispidulin, *p*-synephrine, adipocytes, adipogenesis

## Abstract

Hispidulin is abundant in *Arrabidaea chica*, *Crossostephium chinense*, and *Grindelia argentina*, among others. *p*-Synephrine is the main phytochemical constituent of *Citrus aurantium*. It has been used in combination with various other phytochemicals to determine synergistic effects in studies involving human participants. However, there have been no reports comparing the anti-adipogenic effects of the combination of hispidulin and *p*-synephrine. The current study explores the anti-adipogenic effects of hispidulin alone and in combination with *p*-synephrine in a murine preadipocyte cell line, 3T3-L1. Co-treatment resulted in a greater inhibition of the formation of red-labeled lipid droplets than the hispidulin or *p*-synephrine-alone treatments. Co-treatment with hispidulin and *p*-synephrine also significantly inhibited adipogenic marker proteins, including Akt, mitogen-activated protein kinases, peroxisome proliferator-activated receptor gamma, CCAAT/enhancer-binding protein alpha, glucocorticoid receptor, and CCAAT/enhancer-binding protein β. Although further studies are required to assess the effects of each drug on pharmacokinetic parameters, a combination treatment with hispidulin and *p*-synephrine may be a potential alternative strategy for developing novel anti-obesity drugs.

## 1. Introduction

Adipogenesis is a process by which preadipocytes differentiate into mature adipocytes [1]. In the development of obesity, an increased adipose tissue size results in an increased adipocyte cell size (adipocyte hypertrophy) and adipocyte cell number (adipocyte hyperplasia) [2]. These mechanisms are implicated in childhood obesity and obesity-related metabolic disturbances [3]. In particular, adipocyte hypertrophy is implicated as the main cause of adult-onset obesity [4], whereas adipocyte hyperplasia in adults occurs when existing adipocytes reach a critical size [5].

Phentermine, diethylpropion, phendimetrazine, and mazindol are drugs used to treat obesity by suppressing the appetite and increasing energy expenditure through the regulation of norepinephrine and dopamine metabolism [6,7,8,9]. In addition, Qsymia^®^, an FDA-approved combination drug of phentermine and topiramate, demonstrates the addictive and synergistic effects of the individual components, which have different mechanisms of action to treat obesity [10]. However, the use of these drugs is limited due to critical side effects, such as dizziness, dry mouth, anxiety, insomnia, and increased blood pressure [11]. For these reasons, several studies have attempted to find safe phytochemicals from natural products to develop novel drugs or dietary supplements for weight loss [12]. For instance, curcumin, a major component of *Curcuma longa* (roots), and capsaicin, an alkaloid from *Capsicum annuum* (fruits), showed anti-obesity effects by suppressing 3T3-L1 adipocyte differentiation [13,14]. In addition, research on various natural resources, such as marine organisms and microorganisms, is being actively conducted to develop therapeutics for obesity [15,16]. As such, the development of alternative obesity drugs from natural products has attracted considerable interest.

*p*-Synephrine is the main phytochemical constituent of *Citrus aurantium* (fruits). It is a protoalkaloid, and its chemical structure is similar to ephedrine [17]. The use of ephedrine for weight loss has been banned in some countries, mainly due to adverse effects on the cardiovascular system. Thus, *p*-synephrine has been used to replace ephedrine in dietary supplements [17,18]. Studies have demonstrated the low or negligible toxicity of *p*-synephrine in both in vitro and in vivo experimental models [19,20,21,22]. A study involving human participants showed that the combination of *p*-synephrine with two flavonoids (hesperidin and naringin) increased the thermogenic effects associated with weight loss [23]. In this study, we attempt to identify a flavonoid that exhibits improved effects over other flavonoids (hesperidin and naringin) when treated with *p*-synephrine.

Hispidulin is a flavonoid derived from plants, including *Arrabidaea chica* (leaves), *Crossostephium chinense* (whole plants), *Grindelia argentina* (aerial parts), and *Cirsium japonicum* (whole plants) [24]. It has been reported to exhibit various pharmacological effects, such as anti-fungal, neuroprotective, antioxidant, anti-inflammatory, anti-cancer, and anti-osteoporotic effects [25,26,27,28,29,30]. Accumulated results demonstrate that hispidulin shows low or negligible toxicity in both in vitro and in vivo experimental models [31,32,33]. Recently, an in vitro study indicated that *p*-synephrine and hispidulin suppressed adipogenesis in 3T3-L1 adipocytes [34,35]. The agonists of the gamma-aminobutyric acid type A receptor (GABA_A_-R) expressed in adipose tissues have been shown to be valuable in the treatment of obesity [36]. Hispidulin is known to act as a positive allosteric modulator of the α1,3,5,6β2γ2S GABA_A_-R subtype [37]. One pharmacokinetic study reported a maximum plasma concentration of 2 ng/mL after an oral administration of 46.9 mg *p*-synephrine in humans [38]. Another pharmacokinetic study using a rat model showed a maximum hispidulin plasma concentration of 32 ng/mL after an oral administration of 6 mL/kg *Cirsium japonicum* extract [39]. Both studies suggest that high concentrations of individual compounds cannot be reached in the body by consuming plant-based foods or pure chemical drug substances. A strategy to address this problem is to combine several phytochemical constituents from various plants. In particular, we focused on the common and differing mechanisms of action of *p*-synephrine and hispidulin.

Network pharmacology has developed rapidly based on the concepts of systems biology and polypharmacology. The concept of “multi-drugs–multi-targets” has expanded in terms of existing drug discovery, in which the concept of a “single drug–single target” was previously predominant [40]. In particular, network pharmacology facilitates the prediction of active ingredients and mechanisms of action of natural products composed of various components [41]. A number of recent studies have used network pharmacology to investigate the mechanisms of action of compounds from natural products. For instance, Zhang et al. isolated oxyepiberberine from *Coptis chinensis* (rhizomes) and applied a network pharmacology analysis to identify the mechanism underlying its anti-cancer potential [42]. Cui et al. utilized a network pharmacology approach to understand the anti-inflammatory mechanism of phytochemicals from *Salvia miltiorrhiza* (roots) [43]. As such, network pharmacology plays an important role in overcoming the limitations of studies on conventional natural products by offering a new approach to predict the active ingredients, potential targets, and mechanisms of action.

In this study, we used a network pharmacology-based approach to predict potential targets and mechanisms of action of the anti-obesity effects of *p*-synephrine and hispidulin. We experimentally assessed the anti-obesity effects of *p*-synephrine and hispidulin when used alone and in combination to confirm their additive and synergistic effects when used in combination in 3T3-L1 cells.

## 2. Materials and Methods

### 2.1. Network Pharmacology Analysis

#### 2.1.1. Acquisition of Hispidulin, *p*-Synephrine, and Disease-Related Targets

All the targets of hispidulin and *p*-synephrine were obtained from the PubChem database (http://pubchem.ncbi.nlm.nih.gov/ (accessed on 19 August 2021)) and SwissTargetPrediction database (http://www.swisstargetprediction.ch/ (accessed on 19 August 2021)) [44]. The SMILES of compounds was obtained from the PubChem database and entered into the SwissTargetPrediction database to obtain the predicted targets. In addition, the GeneCards database (http://www.genecards.org/ (accessed on 19 August 2021)) [45] was used to detect the pathological targets of obesity.

#### 2.1.2. Acquisition of Potential Targets

First, duplicates and false-positive targets of the compounds were removed; second, common targets were obtained by comparing with obesity-related targets. These common targets were selected as potential targets. Potential targets were visualized with a Venn diagram using Venny 2.1 (BioinfoGP, Spanish National Biotechnology Centre (CNB-CSIC), Madrid, Sapin) (http://bioinfogp.cnb.csic.es/tools/venny/index.html (accessed on 19 August 2021)) [46]. The DisGeNET database (http://disgenet.org/home/ (accessed on 19 August 2021)) [47] was used to retrieve specific protein class information of potential targets.

#### 2.1.3. Construction and Analysis of Protein–Protein Interaction (PPI) Network

The STRING database (http://string-db.org/ (accessed on 19 August 2021)) [48] was used to obtain PPI networks. Protein interactions with a confidence score ≥0.7 were selected in the designed setting after eliminating duplicates. The resultant data were introduced into Cytoscape (3.8.2) (National Resource for Network Biology (NRNB), Bethesda, MD, USA) to establish the PPI network of potential targets. The PPI network of the potential targets was analyzed using Cytoscape. Three parameters, “degree”, “betweenness centrality”, and “closeness centrality”, were used to assess topological features of nodes in the network. Based on the network analysis, targets within the cut-off values were selected as key targets.

#### 2.1.4. Kyoto Encyclopedia of Genes and Genomes (KEGG) Pathway Enrichment Analysis

KEGG pathway enrichment analysis of the key targets was performed using the DAVID Bioinformatics Resources 6.8 database (http://david.ncifcrf.gov/home.jsp (accessed on 19 August 2021)) [49]. The false discovery rate (FDR) error control method (FDR < 0.05) was used to correct the *p*-value. Finally, a threshold value of *p* < 0.05 was set and signaling pathways were obtained. The KEGG pathway enrichment analysis results were visualized using ImageGP (EHBIO Gene Technology, Beijing, China) (http://www.ehbio.com/ImageGP (accessed on 19 August 2021)).

#### 2.1.5. Construction and Analysis of Compound–Target–Pathway (C–T–P) Networks

The targets associated with this pathway were obtained from the KEGG pathway enrichment analysis. Cytoscape (3.8.2) (NRNB, Bethesda, MD, USA) was used to visualize and analyze the C–T–P network.

### 2.2. Cell Culture and Adipogenic Differentiation

The mouse preadipocyte cell line (3T3-L1) was obtained from the American Type Culture Collection (Manassas, VA, USA) and grown in Dulbecco’s modified Eagle’s medium (DMEM; Cellgro, Manassas, VA, USA) containing 10% bovine calf serum (BCS; Gaithersburg, MD, USA) and 1% penicillin/streptomycin antibiotics (P/S; Gaithersburg, MD, USA). To induce adipogenesis, 3T3-L1 preadipocytes (4 × 10^4^ cells/well) were grown in a 24-well plate for 2 days, and then the culture medium was replaced with the adipogenic differentiation medium containing 0.4 μg/mL dexamethasone (Sigma-Aldrich, St. Louis, MO, USA), 10% fetal bovine serum (FBS; Gaithersburg, MD, USA), 1-methyl-3-isobutylxanthine (IBMX; Sigma-Aldrich, St. Louis, MO, USA), 1% P/S antibiotics, and 5 µg/mL insulin (Sigma-Aldrich, St. Louis, MO, USA). After incubation for 2 days, the culture medium was replaced with DMEM supplemented with 10% FBS, 5 µg/mL insulin, and 1% P/S antibiotics every 2 days. Finally, the culture medium was replaced with DMEM containing 10% FBS and 1% P/S antibiotics, which was changed every 2 days, as previously described [50]. Hispidulin (5, 10, 20, and 40 μM) and *p*-synephrine (5, 10, 20, and 40 μM) were added individually or in combination in the culture medium during adipogenic differentiation. Hispidulin (≥98%) and *p*-synephrine (≥98%) were purchased from Sigma-Aldrich (St. Louis, MO, USA).

### 2.3. Measurement of Cell Viability

The viability of 3T3-L1 preadipocytes was assessed using a tetrazolium salt (WST-1)-based colorimetric assay kit (Ez-Cytox Cell Viability Assay Kit; Daeil Lab Service, Seoul, Korea). The 3T3-L1 preadipocytes (4 × 10^4^ cells/well, 96-well plate) were grown in 10% BCS and 1% P/S antibiotics for 24 h, and then treated with hispidulin (5, 10, 20, and 40 μM) and *p*-synephrine (5, 10, 20, and 40 μM) individually or in combination. After treatment for 24 h, EZ-Cytox reagent was added, and the 3T3-L1 preadipocytes were further incubated for 40 min. The spectrophotometric absorbance was measured using a PowerWave XS microplate reader (BioTek Instruments, Winooski, VT, USA) at 490 nm, as previously described [51].

### 2.4. Oil Red O Staining

On day 8, differentiated cells were fixed with 4% paraformaldehyde solution (Sigma-Aldrich, St. Louis, MO, USA) for 1 h and stained with Oil Red O solution containing 0.5% Oil Red O (ORO; Sigma-Aldrich, St. Louis, MO, USA), 40% distilled water (DW), and 60% isopropanol (Sigma-Aldrich, St. Louis, MO, USA) for 1 h. After washing with DW, lipid droplets stained with ORO were imaged under an inverted microscope at 20× magnification and eluted with 100% isopropanol. The spectrophotometric absorbance was measured on a PowerWave XS microplate reader at 540 nm, as previously described [52].

### 2.5. Western Blot Analysis

On day 8 of cell differentiation, whole cell protein lysates from differentiated cells were prepared, resolved by 10% sodium dodecyl sulfate-polyacrylamide gel electrophoresis, and transferred to nitrocellulose membranes. Target proteins, including phospho-protein kinase B (P-Akt), Akt, phospho-extracellular signal-regulated kinase (P-ERK), ERK, phospho-c-Jun N-terminal kinase (P-JNK), JNK, phospho-P38 (P-P38), P38, peroxisome proliferator-activated receptor gamma (PPAR-γ), CCAAT/enhancer-binding protein alpha (C/EBP-α), C/EBP-β, glucocorticoid receptor (GR), and glyceraldehyde 3-phosphate dehydrogenase (GAPDH), were detected using primary antibodies and horseradish peroxidase-labeled anti-rabbit secondary antibodies (Cell Signaling Technology, Danvers, MA, USA). Target proteins were visualized using ECL Plus Western blotting detection reagents (GE Healthcare, Piscataway, NJ, USA). Protein levels were determined densitometrically using a chemiluminescence system (FUSION Solo, PEQLAB Biotechnologie GmbH, Erlangen, Germany), as previously described [53].

### 2.6. Statistical Analysis

Statistical significance was determined using one-way analysis of variance and multiple comparisons with Bonferroni correction. Statistical significance was set at *p* < 0.05. All analyses were performed using SPSS Statistics ver. 19.0 (SPSS Inc., Chicago, IL, USA).

## 3. Results

### 3.1. Network Pharmacology Analysis

#### 3.1.1. Target Prediction and Screening of Potential Targets

**Figure 1:**
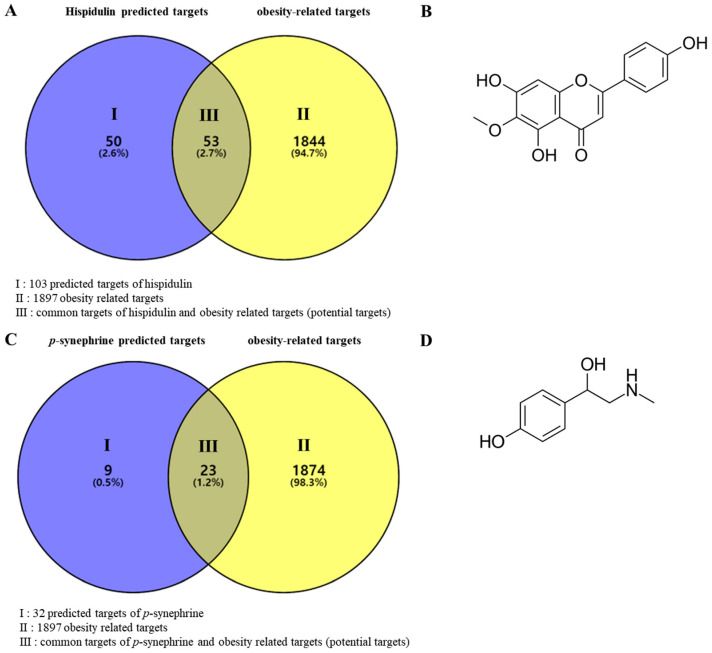
Venn diagrams of predicted targets of compounds and obesity-related targets. (**A**) Venn diagram of hispidulin-predicted targets and obesity-related targets. (**B**) Chemical structure of hispidulin. (**C**) Venn diagram of *p*-synephrine-predicted targets and obesity-related targets. (**D**) Chemical structure of *p*-synephrine.

The SwissTargetPrediction database was used to predict the targets of hispidulin and *p*-synephrine. In data preprocessing, 103 and 32 verified targets of hispidulin and *p*-synephrine, respectively, were screened. In addition, 9489 obesity-related targets were acquired from the GeneCards database, and the relevance score was used as a cut-off value. Based on the relevance score, 1897 obesity-related targets belonging to the top 20% were used for the analysis. As shown in Figure 1, the predicted targets of hispidulin and *p*-synephrine shared 53 and 23 targets, respectively, with obesity-related targets. Thus, these targets were selected as potential targets (Table 1 and Table 2).

#### 3.1.2. Construction of PPI Network

**Figure 2:**
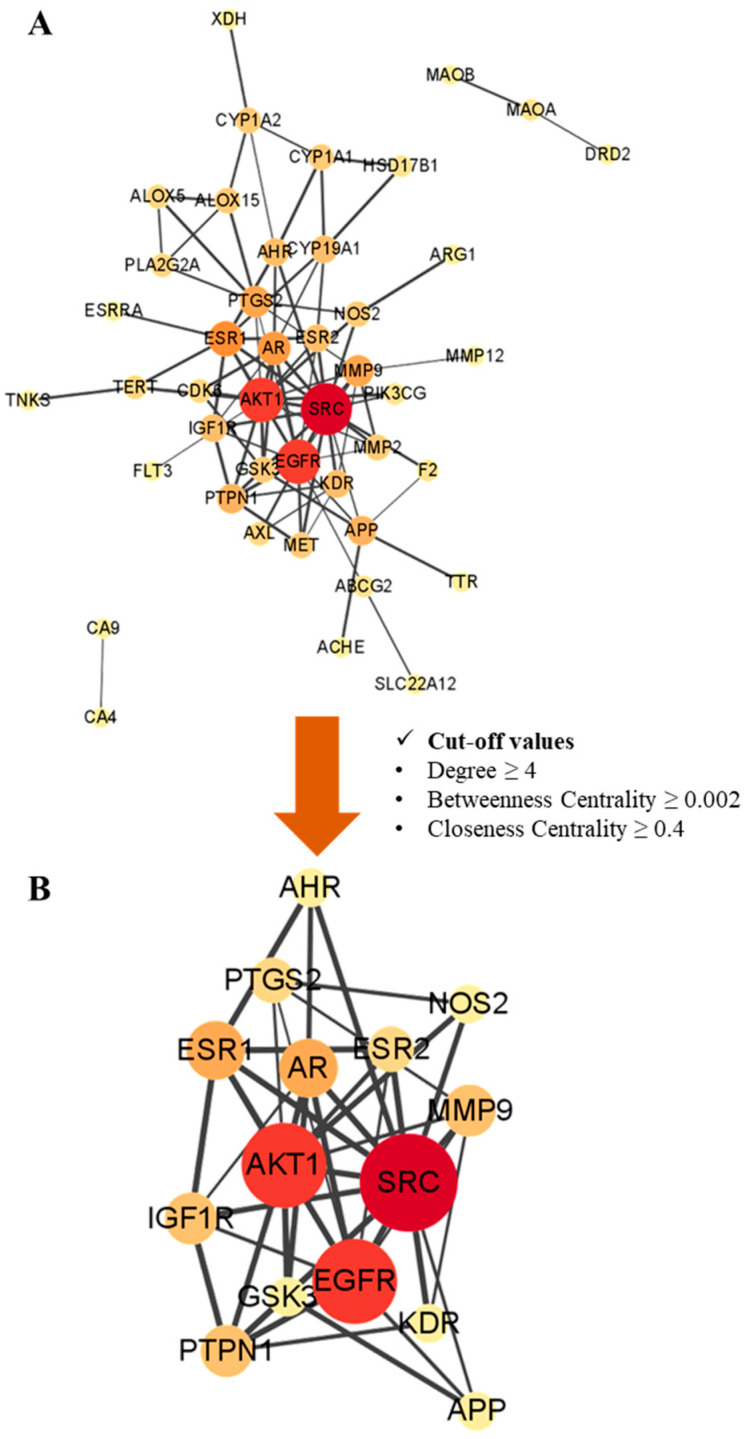
Protein–protein interaction (PPI) network of potential targets and key targets. (**A**) The PPI network of potential anti-obesity target genes of hispidulin. (**B**) The PPI network of the key anti-obesity target genes of hispidulin. The size and the red hue of a node represent its significance within the network.

**Figure 3:**
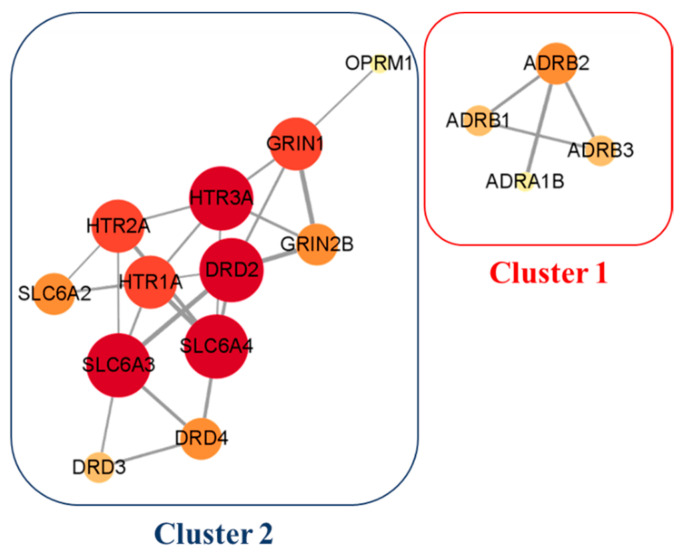
Protein–protein interaction network of potential anti-obesity target genes of *p*-synephrine. The size and red hue of a node represent its significance within the network.

To further explore the interaction between the potential targets, 53 hispidulin anti-obesity potential targets and 23 *p*-synephrine anti-obesity potential targets were put into the STRING database. The PPI networks were placed in the Cytoscape software for a visualization and analysis (Figure 2 and Figure 3). The three parameters, (1) degree, (2) betweenness centrality, and (3) closeness centrality, were applied to analyze the PPI networks. These three parameters indicate the importance and influence of the node in a complex network. The degree (degree centrality) is defined as the number of connections owned by a node [54]. Thus, it is the most straightforward and most intuitive indicator of the importance of a node in the network. The betweenness centrality measures the extent to which a node plays a bridging role in a network. Precisely, it measures the node falls on the shortest path between other pairs of nodes in the network [55]. The closeness centrality is related to the distance between nodes. It is calculated as the average of the shortest path length from the node to every other node in the network [56]. The nodes in the networks represent the target genes, and the edges symbolize the connections between target genes. The size and color of a node indicates the intensity of the degree. Thus, the higher the degree of the target, the larger the node, and the color gradually deepens from yellow to red. The width of the edge designates the grade of the correlation between the targets; the larger the combined score, the higher the binding degree between targets and the thicker the edge. As shown in Figure 2A, the PPI network of hispidulin anti-obesity potential targets consisted of 44 nodes and 90 edges. To identify the key targets among the 44 potential targets, three analytical index cut-off values were applied—degree ≥ 4, betweenness centrality ≥ 0.002, and closeness centrality ≥ 0.4, and a total of 15 targets was identified that satisfied the cut-off values. According to the PPI network analysis of hispidulin anti-obesity targets (Figure 2B), *SRC* (proto-oncogene tyrosine-protein kinase Src), *EGFR* (epidermal growth factor receptor), and *AKT1* (AKT serine/threonine kinase 1) were the top three genes based on the degree (Table 3). The network visualization and analysis were also performed for the 23 *p*-synephrine anti-obesity potential targets. Potential PPI network targets constructed had 16 nodes and 26 edges (Figure 3). As shown in Figure 3, the PPI network of *p*-synephrine anti-obesity potential targets formed two clusters. One of the two clusters was an adrenergic receptor cluster and the other was a dopamine/serotonin receptor cluster. All targets in the two clusters were selected as key targets. The topological analysis results of the *p*-synephrine anti-obesity key targets are listed in Table 4.

#### 3.1.3. KEGG Pathway Enrichment Analysis

**Figure 4:**
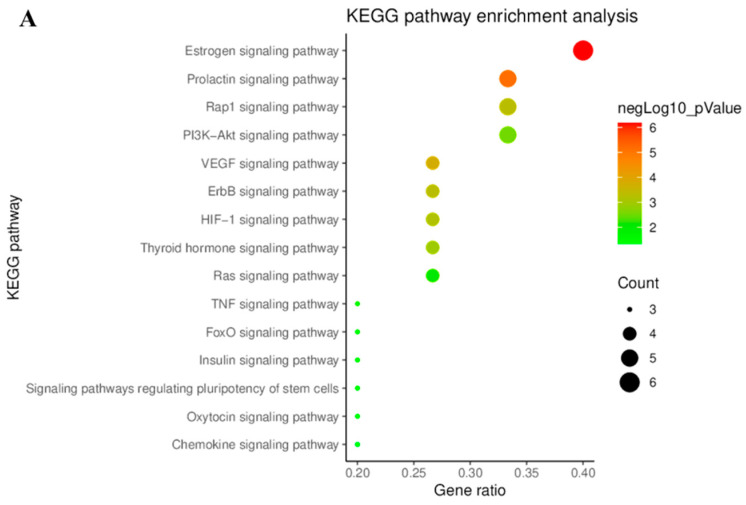

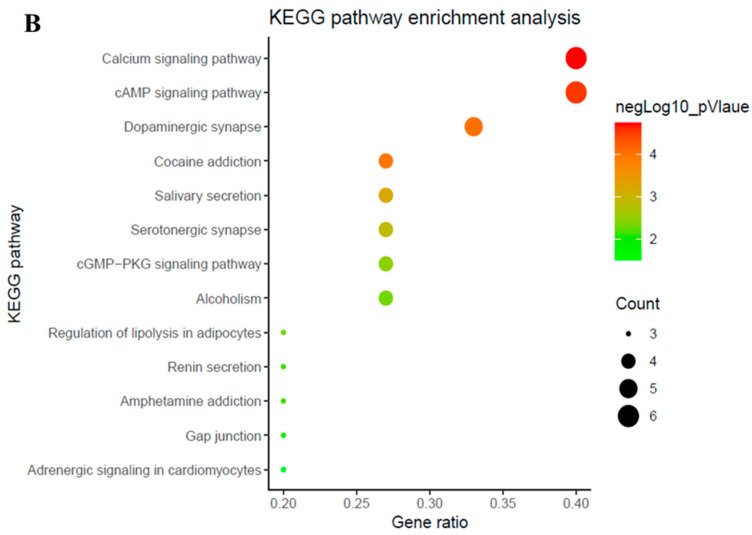
Bubble diagrams of the KEGG pathway enrichment analysis. (**A**) Bubble diagram visualizing KEGG pathway analysis of hispidulin anti-obesity key targets. (**B**) Bubble diagram visualizing KEGG pathway analysis of *p*-synephrine anti-obesity key targets.

The DAVID database was used to identify signaling pathways associated with the key targets of hispidulin and *p*-synephrine. The results of the biological pathways are shown in Figure 4. As shown in Figure 4A, the key anti-obesity targets of hispidulin were primarily related to estrogen, prolactin, CEGF, and Rap1 signaling pathways. In particular, the estrogen signaling pathway exhibited the highest *p*-value. For *p*-synephrine, two pathways, the calcium signaling pathway and the cAMP signaling pathway, showed very high *p*-values.

#### 3.1.4. Construction and Analysis of Compound–Target–Pathway Networks

**Figure 5:**
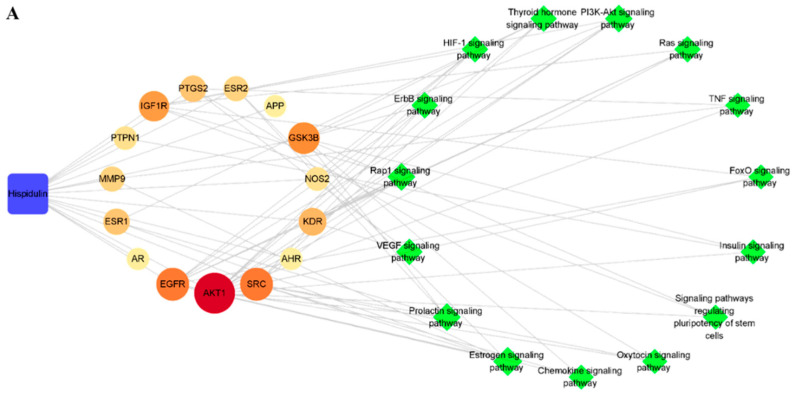

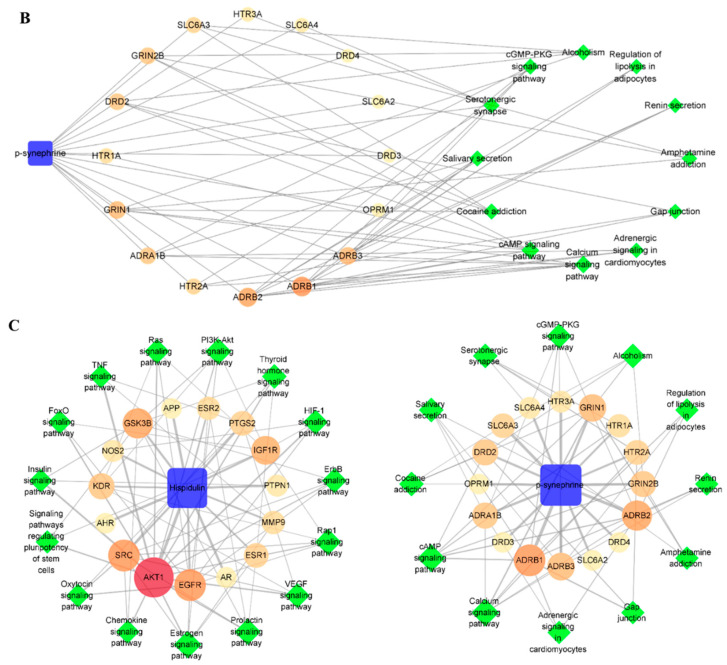
Integrated compound–target–pathway (C–T–P) networks. (**A**) C–T–P network of hispidulin. (**B**) C–T–P network of *p*-synephrine. (**C**) Combination C–T–P network of hispidulin and *p*-synephrine.

An integrative network analysis was performed using Cytoscape to obtain a more comprehensive understanding of the compounds, selected key targets, and pathways related to the two drugs. The C–T–P networks are shown in Figure 5. Blue squares represent compounds, reddish circles represent key targets, and green diamonds represent pathways. The size and color of the circles indicate the degree of each target. Through the network analysis, the parameter degree, betweenness centrality, and closeness centrality were calculated. In the network analysis, the degree indicates the direct influence and importance of the node. Therefore, high degree nodes play important roles in the network.

As shown in Figure 5A, the hispidulin C–T–P network consisted of 31 nodes (1 compound node, 15 key target nodes, and 15 pathway nodes) and 74 edges. Among the key target nodes, *AKT1*, *SRC*, *EGFR*, and *GSK3B* showed high degree values of 15, 9, 9, and 8, respectively. In the pathway nodes, estrogen, prolactin, Rap1, and PI3K-Akt signaling pathways exhibited the degree values of 6, 5, 5, and 5, respectively.

In Figure 5B, the *p*-synephrine C–T–P network formed 1 compound node, 16 key target nodes, 12 pathway nodes, and 63 edges. In particular, *ADRB1*, *ADRB2*, *GRIN1*, and *ADRB3* showed high degree values of 9, 8, 6, and 6, respectively. Among these, *ADRB1*, *ADRB2*, and *ADRB3* were the key targets that clustered in the PPI network analysis. In addition, these targets were connected to the calcium and cAMP signaling pathways, which had the highest degree values among the pathway nodes.

The combination C–T–P network consisted of 60 nodes (2 compound nodes, 31 key target nodes, and 27 pathway nodes) and 137 edges, as shown in Figure 5C. As shown in the combination network, there were no shared key targets or pathways among the predicted key targets and pathways. These results suggest that hispidulin and *p*-synephrine might exhibit anti-obesity effects through different mechanisms of action.

### 3.2. Inhibitory Effects of Hispidulin and *p*-Synephrine on Adipogenesis in 3T3-L1 Preadipocytes

**Figure 6:**
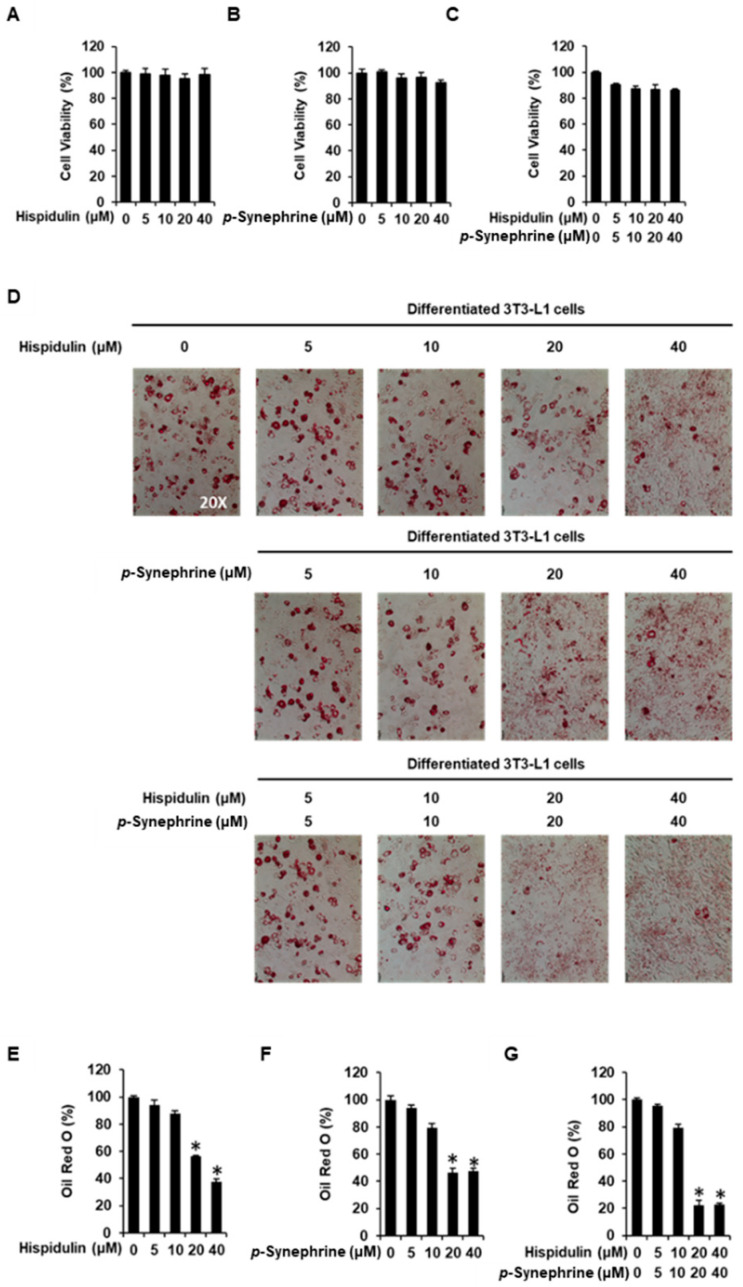
Inhibitory effects of hispidulin and *p*-synephrine on adipogenesis in 3T3L-1 preadipocytes. Effect of (**A**) hispidulin, (**B**) *p*-synephrine, and (**C**) combination of hispidulin and *p*-synephrine on the viability of 3T3L-1 preadipocytes for 24 h by Ez-Cytox cell viability assay. (**D**) Images of the Oil Red O staining of differentiated 3T3L-1 cells imaged under an inverted microscope at 20× magnification on day 8 after treatment with hispidulin and/or *p*-synephrine. (**E**–**G**) Quantification of Oil Red O staining expressed as the percentage of the untreated control (*n* = 3 independent experiments, * *p* < 0.05, Kruskal–Wallis nonparametric test). Data are presented as the mean ± SEM.

The cytotoxicity of hispidulin, *p*-synephrine, and co-treatment with hispidulin and *p*-synephrine in 3T3-L1 preadipocytes was evaluated using the Ez-Cytox cell viability assay kit. The cell viability assay showed that concentrations up to 40 µM hispidulin and 40 µM *p*-synephrine, and the co-treatment with up to 40 µM hispidulin and 40 µM *p*-synephrine, did not affect the viability of 3T3-L1 preadipocytes after 24 h of incubation (Figure 6A–C).

The inhibitory effects of hispidulin and *p*-synephrine at non-toxic concentrations on adipogenesis were determined using Oil Red O staining of 3T3-L1 preadipocytes (Figure 6D). Treatment with 20 µM and 40 µM hispidulin inhibited the differentiation of 3T3-L1 preadipocytes into mature adipocytes. The cells treated with 20 µM and 40 µM hispidulin showed a slight but not significant inhibition (56.63 ± 0.53% and 37.75 ± 1.81% reduction, respectively) of the formation of red-labeled lipid droplets. Similarly, treatment with 20 µM and 40 µM *p*-synephrine inhibited the differentiation of 3T3-L1 preadipocytes into mature adipocytes. The cells treated with 20 µM and 40 µM *p*-synephrine showed a slight but not significant inhibition (46.24 ± 4.53% and 47.59 ± 2.66% reduction, respectively) of the formation of red-labeled lipid droplets. However, co-treatment with 20 µM and 40 µM hispidulin and 20 µM and 40 µM *p*-synephrine resulted in a greater inhibition of the formation of red-labeled lipid droplets than the hispidulin or *p*-synephrine-alone treatment. Co-treatment with hispidulin (20 µM and 40 µM) and *p*-synephrine (20 µM and 40 µM) significantly inhibited the differentiation of 3T3-L1 preadipocytes into mature adipocytes. The cells treated with equal concentrations of hispidulin and *p*-synephrine (20 µM and 40 µM) showed a significant inhibition (22.28 ± 4.04% and 22.96 ± 1.11% reduction, respectively) of the formation of red-labeled lipid droplets (Figure 6E–G).

### 3.3. Effect of Hispidulin and *p*-Synephrine on the Expression of Proteins Involved in Adipogenesis in Differentiated 3T3L-1 Cells

**Figure 7:**
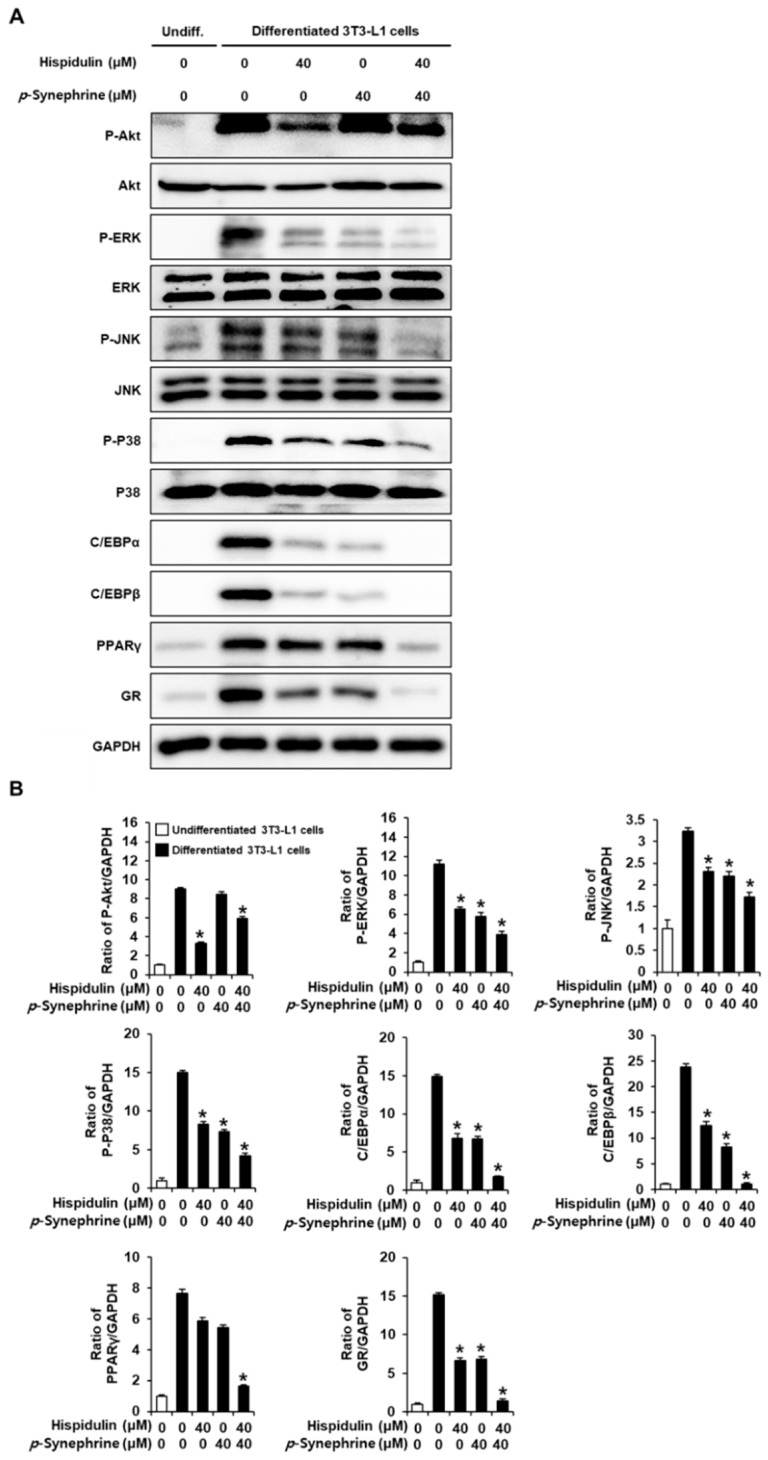
Inhibitory effects of hispidulin and *p*-synephrine on the expression of adipogenesis-related proteins in differentiated 3T3L-1 cells. (**A**) The protein expression of phospho-Akt (P-Akt), Akt, phospho-extracellular signal-regulated kinase (P-ERK), ERK, phospho-c-Jun-N-terminal kinase (P-JNK), JNK, phospho-P38 (P-P38), P38, peroxisome proliferator-activated receptor gamma (PPARγ), CCAAT/enhancer-binding protein alpha (C/EBPα), C/EBPβ, glucocorticoid receptor (GR), and glyceraldehyde 3-phosphate dehydrogenase (GAPDH) in differentiated 3T3L-1 cells on day 8 after treatment with hispidulin and/or *p*-synephrine. (**B**) Analysis of the ratios of the band intensities of P-ERK, P-JNK, P-P38, PPARγ, C/EBPα, C/EBPβ, and GR in the treated cells compared with those in the untreated differentiated 3T3L-1 cells (*n* = 3 independent experiments, * *p* < 0.05, Kruskal–Wallis nonparametric test). Data are presented as the mean ± SEM.

To examine how hispidulin and *p*-synephrine inhibited adipogenesis in 3T3-L1 cells, we used the Western blot analysis to examine the expression of adipogenic marker proteins, including Akt, ERK, JNK, P38, PPARγ, C/EBPα, GR, and C/EBPβ (Figure 7A). Treatment with either 40 µM hispidulin or 40 µM *p*-synephrine slightly inhibited the expression of phospho-ERK, phospho-JNK, phospho-P38, PPARγ, C/EBPα, GR, and C/EBPβ in differentiated 3T3L-1 cells compared with the untreated controls. Co-treatment with hispidulin and *p*-synephrine further suppressed the expression of these proteins. In particular, after treatment with hispidulin, the expression of P-Akt was significantly suppressed, whereas *p*-synephrine had no effect on the expression of P-Akt compared with the untreated differentiated 3T3L-1 cells. Co-treatment with hispidulin and *p*-synephrine slightly suppressed the expression of P-Akt (Figure 7A,B). This suggested that the co-treatment of hispidulin and *p*-synephrine was effective in decreasing adipogenic marker proteins during the eight-day adipocyte differentiation period.

## 4. Discussion

In this study, we applied a network pharmacology analysis to predict the anti-obesity mechanism of action of hispidulin and *p*-synephrine. Through a network pharmacology analysis, the anti-obesity effect of hispidulin was predicted to act on estrogen, prolactin, Rap1, and PI3K-Akt signaling pathways by targeting *AKT1*, *SRC*, *EGFR*, and *GSK3B*. Previous studies have reported that these signaling pathways are related to obesity or adipocyte metabolism [57,58,59,60]. In addition, *p*-synephrine was predicted to exert its anti-obesity effect via calcium and cAMP signaling pathways by targeting adrenergic receptors, *ADRB1*, *ADRB2*, and *ADRB3*. In particular, a number of studies has provided evidence regarding the relationship between β3-adrenergic receptors (*ADRB3*) and obesity [61,62,63]. Moreover, recent studies have shown that the calcium signaling pathway specifically plays a key role in reducing obesity by enhancing energy consumption and promoting adipocyte differentiation and metabolism [64,65,66,67]. Based on the results of previous studies, the network pharmacology analysis in the present study predicted a feasible possible mechanism of action of hispidulin and *p*-synephrine against obesity.

Furthermore, the results of the combination network analysis of the two compounds showed completely different targets and pathways, which suggests that combination treatment with hispidulin and *p*-synephrine might exhibit additive and synergistic effects through different mechanisms of action. Among the commercially available diet drugs, Qsymia^®^ (phentermine/topiramate) and Contrave^®^ (naltrexone/bupropion) are the combinations of two drugs with different mechanisms of action [10,68]. These drugs show a stronger appetite suppressant effect than single drugs through the additive and synergistic effects of the combined components with different mechanisms of action. Based on this evidence, the combination treatment of hispidulin and *p*-synephrine has a potential to show stronger effects against obesity than when used alone. Therefore, additional experiments were performed to verify the results of the network pharmacology analysis and further evaluate the efficacy of hispidulin and *p*-synephrine in single and combination therapies.

Both compounds have already been reported to be effective against adipogenesis in 3T3-L1 cells. A previous study showed that hispidulin at 40 µM exhibited a maximal inhibitory effect (46% reduction) on the formation of red-labeled lipid droplets in 3T3-L1 cells. However, anti-adipogenic effects examined in this study only focused on the protein expression of PPARγ, C/EBPα, and adiponectin [35]. In the same cell lines, *p*-synephrine at 10 µM exhibited a maximal inhibitory effect (26% reduction) on the formation of red-labeled lipid droplets via the regulation of Akt, glycogen synthase kinase 3β (GSK3β), β-catenin, PPARγ, C/EBPα, fatty acid-binding protein 4 (aP2), and glycogen synthase (GS) [34]. However, the detailed mechanisms underlying the anti-adipogenic effects of hispidulin and *p*-synephrine are not yet completely clear.

The inhibitory effect of hispidulin or *p*-synephrine on the formation of red-labeled lipid droplets reported in previous studies is in line with our study. In the present study, co-treatment with hispidulin and *p*-synephrine caused a greater inhibition of the differentiation of 3T3-L1 preadipocytes than hispidulin or *p*-synephrine alone. In this regard, although we did not test the two compounds at higher concentrations, it is expected that concentrations of 40 µM or higher will further inhibit adipogenesis. However, high concentrations of hispidulin or *p*-synephrine at the cellular level in the body may not be possible when ingested through plant-based foods or as pure chemical drugs [38,39]. In addition, there are no definitive studies on the toxicity of hispidulin or *p*-synephrine at high concentrations. Thus, combining hispidulin and *p*-synephrine at low concentrations may be a potential alternative strategy to prevent obesity via consuming plant-based foods or pure chemical drugs.

Subsequently, a mechanistic study was conducted to observe the changes in the levels of adipogenic marker proteins, including PPARγ and C/EBPα, which were highlighted by two previous studies on the effects of hispidulin or *p*-synephrine [34,35]. The anti-adipogenic effect of the combination of hispidulin and *p*-synephrine was accompanied by a decreased protein expression of PPARγ and C/EBPα. These results were consistent with those of the previous studies. PPARγ and C/EBPα are important transcription factors in the terminal differentiation of adipocytes, and their cross-regulation is important in accumulating and storing lipids. In addition to the accumulation and storage of lipids, PPARγ and C/EBPα are important in promoting and maintaining a fully differentiated state in adipocytes [69,70].

Additionally, the combination of hispidulin and *p*-synephrine resulted in a decreased protein expression of the transcription factor C/EBPβ, which plays a principal role in orchestrating early steps of adipogenesis [71]. During the early stage of adipogenesis, the nuclear localization of C/EBPβ is mediated by the activation of ERK, P38, and GR in response to adipogenic stimuli [72,73,74]. In addition, glucocorticoid hormones affect adipocyte differentiation and the maintenance of adipogenic genes by binding to GR, a ligand-activated transcription factor [75,76]. It has been previously shown that JNK is responsible for the transcriptional activity of PPARγ [77,78]. As little is known about the role of JNK in adipocyte differentiation, its potential as a target appears to be currently limited. In the present study, the combination of hispidulin and *p*-synephrine compared to hispidulin or *p*-synephrine caused a stronger inhibition of MAPKs (ERK, JNK, and P38) and GR. These results indicate that hispidulin and *p*-synephrine share a common mechanism in regulating adipogenesis. In particular, after treatment with hispidulin, the phosphorylation of Akt was significantly suppressed, whereas *p*-synephrine had no effect on the phosphorylation of Akt compared with the untreated differentiated 3T3L-1 cells. Co-treatment with hispidulin and *p*-synephrine slightly suppressed Akt phosphorylation. These results suggested that the mechanisms of action of the two compounds had both different and common features. Thus, the target that *p*-synephrine does not affect may be compensated for by co-treatment with hispidulin.

Taken together, the combination of hispidulin and *p*-synephrine significantly inhibited adipocyte differentiation by inhibiting PPARγ and C/EBPα via the regulation of C/EBPβ, GR, and MAPKs (ERK, JNK, and P38) during the differentiation of 3T3-L1 adipocytes. Our results may offer an invaluable scientific experimental basis for the application of the combination of hispidulin and *p*-synephrine for the development of novel anti-obesity drugs. In future, studies identifying pharmacokinetic drug–drug interactions using animal models will be required. In addition, selecting pharmacopuncture as the injection method solves the problem of the concentration of phytochemicals at the physiological level and their stability. Pharmacopuncture is a new method of acupuncture with the injection of chemical ingredients from herbal medicine to the acupoints on the abdomen. Its effect could be observed immediately after injection because chemical ingredients are absorbed directly without going through the gastrointestinal tract. Thus, it is easy to adjust the dosage [79]. Further in vivo studies using pharmacopuncture with standardized methodology should be performed to evaluated the anti-obesity effect of hispidulin and *p*-synephrine.

## 5. Conclusions

In this study, we predicted the mechanisms underlying the anti-obesity effects of hispidulin and *p*-synephrine using a network pharmacology analysis. *AKT1*, *SRC*, *EGFR*, and *GSK3B* were identified as key anti-obesity target genes of hispidulin, and estrogen, prolactin, Rap1, and PI3K-Akt signaling pathways were predicted to be involved in the anti-obesity effects of hispidulin. For *p*-synephrine, adrenergic receptors were predicted as key target genes, and calcium and cAMP signaling pathways were predicted to be associated downstream signaling pathways. Our study revealed that the combination treatment with hispidulin and *p*-synephrine performed better than separate treatments with each compound in suppressing adipogenesis. This additive effect was related to the significant inhibition of protein expression, including MAPKs (ERK, ERK, JNK, and P38), C/EBPα, C/EBPβ, PPARγ, and GR. Specifically, as predicted, the phosphorylation of Akt was suppressed after treatment with hispidulin only. Although further studies are required to assess the pharmacokinetic interactions of the drugs, the combination treatment with hispidulin and *p*-synephrine may be a potential alternative strategy for developing novel anti-obesity drugs.

## Figures and Tables

**Table 1:** Potential targets of hispidulin.

No.	Uniprot ID	Gene	Relevance Score	Target	Protein Class
1	P18031	*PTPN1*	12.186	protein tyrosine phosphatase non-receptor type 1	-
2	P31749	*AKT1*	11.022	AKT serine/threonine kinase 1	Kinase
3	P14679	*TYR*	10.667	tyrosinase	Enzyme
4	P03372	*ESR1*	10.026	estrogen receptor 1	Nuclear receptor
5	P11511	*CYP19A1*	9.307	cytochrome P450 family 19 subfamily A member 1	Enzyme
6	P14416	*DRD2*	8.109	dopamine receptor D2	G-protein coupled receptor
7	P08069	*IGF1R*	7.564	insulin-like growth factor 1 receptor	Kinase
8	P00734	*F2*	6.902	coagulation factor II, thrombin	Enzyme
9	P14780	*MMP9*	6.393	matrix metallopeptidase 9	Enzyme
10	P10275	*AR*	5.830	androgen receptor	Nuclear receptor
11	P21397	*MAOA*	5.226	monoamine oxidase A	-
12	P09917	*ALOX5*	5.000	arachidonate 5-lipoxygenase	Enzyme
13	O14746	*TERT*	4.864	telomerase reverse transcriptase	Enzyme
14	P05177	*CYP1A2*	4.092	cytochrome P450 family 1 subfamily A member 2	Enzyme
15	P12931	*SRC*	4.045	SRC proto-oncogene, non-receptor tyrosine kinase	Kinase
16	P11474	*ESRRA*	3.994	estrogen-related receptor alpha	Nuclear receptor
17	P08253	*MMP2*	3.992	matrix metallopeptidase 2	Enzyme
18	P30542	*ADORA1*	3.818	adenosine A1 receptor	G-protein coupled receptor
19	P15121	*AKR1B1*	3.658	aldo-keto reductase family 1 member B	Enzyme
20	P00533	*EGFR*	3.653	epidermal growth factor receptor	Kinase
21	P14061	*HSD17B1*	3.553	hydroxysteroid 17-beta dehydrogenase 1	Enzyme
22	P35869	*AHR*	3.423	aryl hydrocarbon receptor	Transcription factor
23	P35372	*OPRM1*	3.300	opioid receptor mu 1	G-protein coupled receptor
24	P35228	*NOS2*	3.298	nitric oxide synthase 2	-
25	P35354	*PTGS2*	3.209	prostaglandin-endoperoxide synthase 2	Enzyme
26	P08581	*MET*	3.161	MET proto-oncogene, receptor tyrosine kinase	Kinase
27	Q92731	*ESR2*	3.099	estrogen receptor 2	Nuclear receptor
28	P48736	*PIK3CG*	2.973	phosphatidylinositol-4,5-bisphosphate 3-kinase catalytic subunit gamma	Kinase
29	P51955	*NEK2*	2.886	NIMA related kinase 2	Kinase
30	P22748	*CA4*	2.821	carbonic anhydrase 4	-
31	Q9UNQ0	*ABCG2*	2.640	ATP binding cassette subfamily G member 2 (Junior blood group)	Transporter
32	P05089	*ARG1*	2.614	arginase 1	Enzyme
33	P08183	*ABCB1*	2.514	ATP binding cassette subfamily B member 1	Transporter
34	P49841	*GSK3B*	2.512	glycogen synthase kinase 3 beta	Kinase
35	P22303	*ACHE*	2.449	acetylcholinesterase (Cartwright blood group)	Enzyme
36	O95271	*TNKS*	2.335	tankyrase	-
37	P04798	*CYP1A1*	2.319	cytochrome P450 family 1 subfamily A member 1	Enzyme
38	P02766	*TTR*	2.317	transthyretin	Transporter
39	P35968	*KDR*	2.128	kinase insert domain receptor	Kinase
40	P05067	*APP*	2.119	amyloid beta precursor protein	Enzyme modulator
41	P14555	*PLA2G2A*	2.057	phospholipase A2 group IIA	Enzyme
42	Q16875	*PFKFB3*	1.902	6-phosphofructo-2-kinase/fructose-2,6-biphosphatase 3	Kinase
43	P47989	*XDH*	1.866	xanthine dehydrogenase	Enzyme
44	P04745	*AMY1A*	1.716	amylase alpha 1A	-
45	P39900	*MMP12*	1.685	matrix metallopeptidase 12	Enzyme
46	P27338	*MAOB*	1.649	monoamine oxidase B	-
47	Q00534	*CDK6*	1.574	cyclin dependent kinase 6	Kinase
48	P36888	*FLT3*	1.567	fms-related receptor tyrosine kinase 3	Kinase
49	Q96S37	*SLC22A12*	1.554	solute carrier family 22 member 12	Transporter
50	Q16790	*CA9*	1.553	carbonic anhydrase 9	-
51	P16050	*ALOX15*	1.520	arachidonate 15-lipoxygenase	Enzyme
52	P30530	*AXL*	1.459	AXL receptor tyrosine kinase	Kinase
53	P00918	*CA2*	1.444	carbonic anhydrase 2	-

**Table 2:** Potential targets of *p*-synephrine.

No.	Uniprot ID	Gene	Relevance Score	Target	Protein Class
1	P18031	*PTPN1*	12.186	protein tyrosine phosphatase non-receptor type 1	-
2	P31749	*AKT1*	11.022	AKT serine/threonine kinase 1	Kinase
3	P14679	*TYR*	10.667	tyrosinase	Enzyme
4	P03372	*ESR1*	10.026	estrogen receptor 1	Nuclear receptor
5	P11511	*CYP19A1*	9.307	cytochrome P450 family 19 subfamily A member 1	Enzyme
6	P14416	*DRD2*	8.109	dopamine receptor D2	G-protein coupled receptor
7	P08069	*IGF1R*	7.564	insulin-like growth factor 1 receptor	Kinase
8	P00734	*F2*	6.902	coagulation factor II, thrombin	Enzyme
9	P14780	*MMP9*	6.393	matrix metallopeptidase 9	Enzyme
10	P10275	*AR*	5.830	androgen receptor	Nuclear receptor
11	P21397	*MAOA*	5.226	monoamine oxidase A	-
12	P09917	*ALOX5*	5.000	arachidonate 5-lipoxygenase	Enzyme
13	O14746	*TERT*	4.864	telomerase reverse transcriptase	Enzyme
14	P05177	*CYP1A2*	4.092	cytochrome P450 family 1 subfamily A member 2	Enzyme
15	P12931	*SRC*	4.045	SRC proto-oncogene, non-receptor tyrosine kinase	Kinase
16	P11474	*ESRRA*	3.994	estrogen-related receptor alpha	Nuclear receptor
17	P08253	*MMP2*	3.992	matrix metallopeptidase 2	Enzyme
18	P30542	*ADORA1*	3.818	adenosine A1 receptor	G-protein coupled receptor
19	P15121	*AKR1B1*	3.658	aldo-keto reductase family 1 member B	Enzyme
20	P00533	*EGFR*	3.653	epidermal growth factor receptor	Kinase
21	P14061	*HSD17B1*	3.553	hydroxysteroid 17-beta dehydrogenase 1	Enzyme
22	P35869	*AHR*	3.423	aryl hydrocarbon receptor	Transcription factor
23	P35372	*OPRM1*	3.300	opioid receptor mu 1	G-protein coupled receptor

**Table 3:** Hispidulin anti-obesity key targets identified based on PPI network topological analysis.

No.	Uniprot ID	Gene	Degree	Betweenness Centrality	Closeness Centrality
1	P12931	*SRC*	12	0.277	0.875
2	P31749	*AKT1*	10	0.167	0.778
3	P00533	*EGFR*	10	0.126	0.778
4	P10275	*AR*	6	0.047	0.636
5	P03372	*ESR1*	6	0.025	0.636
6	P14780	*MMP9*	5	0.025	0.609
7	P18031	*PTPN1*	5	0.017	0.609
8	P08069	*IGF1R*	5	0.008	0.609
9	P35354	*PTGS2*	4	0.007	0.560
10	Q92731	*ESR2*	4	0.000	0.583
11	P05067	*APP*	3	0.010	0.560
12	P49841	*GSK3B*	3	0.007	0.538
13	P35228	*NOS2*	3	0.004	0.560
14	P35968	*KDR*	3	0.003	0.538
15	P35869	*AHR*	3	0.002	0.538

**Table 4:** Hispidulin anti-obesity key targets identified based on PPI network topological analysis.

No.	Uniprot ID	Gene	Degree	Betweenness Centrality	Closeness Centrality
1	P14416	*DRD2*	5	0.256	0.647
2	P23975	*SLC6A3*	5	0.211	0.611
3	P31645	*SLC6A4*	5	0.120	0.611
4	P46098	*HTR3A*	5	0.159	0.611
5	Q05586	*GRIN1*	4	0.188	0.550
6	P08908	*HTR1A*	4	0.053	0.579
7	P28223	*HTR2A*	4	0.053	0.579
8	Q13224	*GRIN2B*	3	0.006	0.524
9	P07550	*ADRB2*	3	0.667	1.000
10	P21917	*DRD4*	3	0.029	0.458
11	P08913	*SLC6A2*	3	0.017	0.500
12	P13945	*ADRB3*	2	0.000	0.750
13	P08588	*ADRB1*	2	0.000	0.750
14	P35462	*DRD3*	2	0.000	0.423
15	P35368	*ADRA1B*	1	0.000	0.600
16	P35372	*OPRM1*	1	0.000	0.367

## Data Availability

Data is contained within the article.
